# Draft Genome Sequence of the *bla*_OXA-436_- and *bla*_NDM-1_-Harboring *Shewanella putrefaciens* SA70 Isolate

**DOI:** 10.1128/genomeA.00644-17

**Published:** 2017-07-20

**Authors:** Robert F. Potter, Alaric W. D’Souza, Meghan A. Wallace, Angela Shupe, Sanket Patel, Danish Gul, Jennie H. Kwon, Saadia Andleeb, Carey-Ann D. Burnham, Gautam Dantas

**Affiliations:** a Center for Genome Sciences and Systems Biology, Washington University School of Medicine, St. Louis, Missouri, USA; b Department of Pathology and Immunology, Washington University School of Medicine, St. Louis, Missouri, USA; c Atta ur Rahman School of Applied Biosciences, National University of Sciences and Technology Islamabad, Islamabad, Pakistan; d Division of Infectious Diseases, Washington University School of Medicine, St. Louis, Missouri, USA; e Department of Pediatrics, St. Louis Children’s Hospital, St. Louis, Missouri, USA; f Department of Molecular Microbiology, Washington University School of Medicine, St. Louis, Missouri, USA; g Department of Biomedical Engineering, Washington University in St. Louis, St. Louis, Missouri, USA

## Abstract

We sequenced a carbapenem-resistant *Shewanella putrefaciens* isolate cultured from the sink handle of a Pakistan hospital room. Assembly annotation indicates that the isolate has a chromosomal *bla*_OXA-436_ carbapenemase and a plasmid-borne *bla*_NDM-1_ gene. To our knowledge, this is the first report of a *Shewanella* species harboring *bla*_NDM_.

## GENOME ANNOUNCEMENT

*Shewanella putrifaciens* is an infrequent human pathogen associated with opportunistic infections (1–3). We isolated *S. putrefaciens* from the sink handle of an intensive care unit room from a tertiary care hospital in Pakistan using the Eswab collection device and transport system (Becton, Dickinson, and Company). The isolate was recovered on MacConkey agar with 5 μg/mL cefotaxime (Hardy Diagnostics) and identified with a confidence value of 99.9% using VITEK matrix-assisted laser desorption ionization–time of flight mass spectrometry (MALDI-TOF MS) with library version v2.3.3 (bioMérieux, Durham, NC). Antimicrobial susceptibility testing using Kirby-Bauer disk diffusion per Clinical and Laboratory Standards Institute M2 guidelines demonstrated an aztreonam zone size of 30 mm, ceftazidime zone size of 6 mm, meropenem zone size of 6 mm, and imipenem zone size of 6 mm. Using *Enterobacteriaceae*, *Pseudomonas aeruginosa*, or *Aeromonas* sp. guidelines, the isolate is considered aztreonam susceptible but ceftazidime, meropenem, and imipenem resistant, a resistance profile suggestive of a Class B carbapenemase. The isolate was also positive for phenotypic production of a carbapenemase via the carbapenem inactivation method (4). To identify known carbapenemase genes in *S. putrefaciens*, we extracted genomic DNA with the Bacteremia kit (MoBIO, Carlsbad, CA), used 0.5 ng of DNA to make a Nextera XT Illumina sequencing library (5), and performed whole-genome sequencing on an Illumina NextSeq high-output sequencer to obtain 1,454,810 2 × 150 bp reads.

We processed the sequence data on the High Throughput Computing Facility 2250 processor computing cluster at Washington University, St. Louis School of Medicine. Nextera indexes and sequencing primers were trimmed from forward and reverse reads using Trimmomatic, and human genome sequence reads, which may bleed through from libraries on the same lane, were removed using DeconSeq (6, 7). The resulting 1,452,994 paired-end reads were assembled with SPAdes (8). QUAST determined that the assembly contained 302 contigs (354,418 bp largest contig) and 70,827 bp *N*_50_ for contigs with >0 nucleotides (9). We annotated contigs with lengths >500 bp, representing 5,110,564 bp, using RAST (10). RAST identified 10 rRNA genes, 71 tRNA genes, and 4,522 CDS. Fifty percent of CDS have annotated functional systems. We identified antibiotic resistance genes with Resfams, finding two class D β-lactamases and two class B β-lactamases (11). BLASTp of the amino acid sequences against the NCBI nonredundant protein database (accessed 14 March 2017) confirmed these genes as *bla*_OXA-436_ (99% identity), *bla*_OXA-10_, a metallo-fold hydrolase, and *bla*_NDM-1_ (all 100% identity). OXA-436 (GenBank accession no. KT959103) and NDM-1 can hydrolyze carbapenems, implicating them as the mechanism of carbapenem resistance (12). BLASTn of the *bla*_NDM-1_ contig against the nonredundant nucleotide database (accessed on 14 March 2017) returned 100% identity to *bla*_NDM-1_ containing IncN type plasmids from *Escherichia coli* (KJ440076) and *Klebsiella pneumoniae* (KJ440075) (13). The *bla*_OXA-436_ carbapenemase contig had the highest BLASTn alignment score to the complete genome of *Shewanella* sp. MR-7 (CP000444).

This *Shewanella putrefaciens* genome documents the diversity of host species possessing *bla*_NDM-1_. Identification of carbapenemases in this isolate supports the importance of antimicrobial susceptibility testing in infrequent pathogens. Importantly, *Shewanella* spp*.* could represent potential reservoirs for horizontally transferred antimicrobial resistance genes within the health care environment.

### Accession number(s).

This whole-genome shotgun project has been deposited at DDBJ/ENA/GenBank under the accession no. NGZL00000000. The version described in this paper is NGZL01000000.
